# Supplementary material for: Is Antimicrobial Dosing Adjustment Associated with Better Outcomes in Patients with Severe Obesity and Bloodstream Infections? An Exploratory Study
Source: Antibiotics (Basel). 2020 Oct 16;9(10):707. doi: 10.3390/antibiotics9100707 (PMC7602836; doi:10.3390/antibiotics9100707)
Supplement: Supplementary file 1 [file antibiotics-09-00707-s001.pdf]

Is antimicrobial dosing adjustment associated with better outcomes in patients with severe obesity and bloodstream infections? An exploratory study

Sirard et al. 2020

Supporting Information

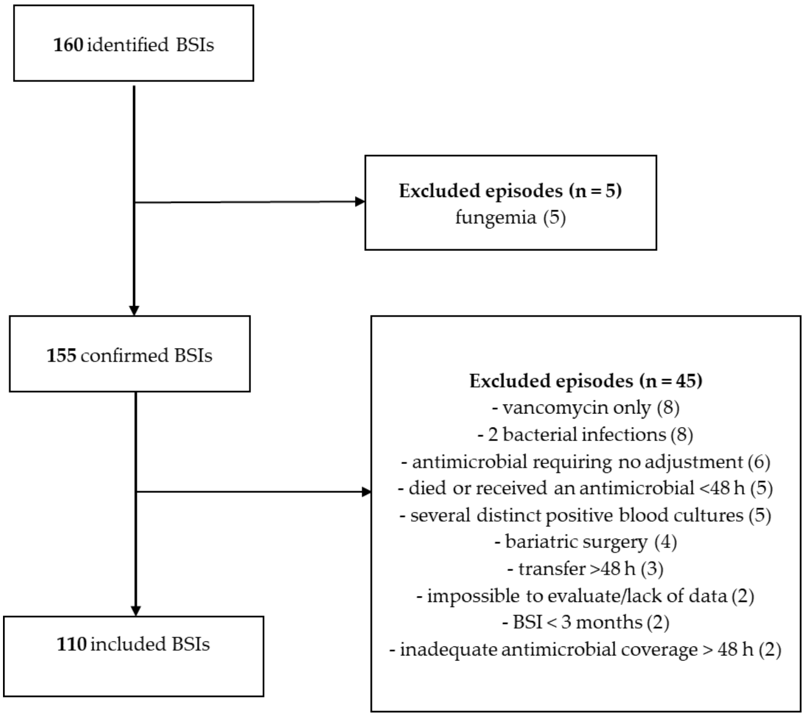

Figure S1. Flowchart of included and excluded episodes of bloodstream infections.

Table S1. Variables related to the appropriateness according to the period of admission.

**Variables**	**pre APSS (2005-2010) *n* = 51 (46%)**	**early APSS (2010-2012) *n* = 19 (17%)**	**late APSS (2013-2015) *n* = 40 (36%)**	**Total cohort N = 110**
Appropriateness category				
Good (80%-100 %)	17 (33)	9 (47)	21 (53)	47 (43)
Moderate (20%-79 %)	15 (29)	5 (26)	11 (28)	31 (28)
Poor (0%-19 %)	19 (37)	5 (26)	8 (20)	32 (29)
Appropriateness of the treatment, % (median, IQR)	27 (12-86)	60 (9-97)*	84 (35-100)*	58 (17-97)
Therapy 100% unadjusted	9 (18)	4 (21)	4 (10)	17 (16)
No of inadequate days (median, IQR)	11 (6.5-14)	10 (3-13)	5 (1-13)	10 (3-14)
First prescription inadequate	30 (59)	11 (58)	25 (63)	66 (60)
First dose inadequate	15 (29)	7 (37)	15 (38)	37 (34)
No of prescriptions (mean ± SD)	3.98 ± 1.73	4.63 ± 2.09	4.58 ± 1.60	4.31 ± 1.76
No. inadequate prescriptions (mean ± SD)	2.35 ± 1.38	2.58 ± 1.12	1.83 ± 0.98	2.2 ± 1.23
No. antimicrobials (mean ± SD)	2.90 ± 1.25	2.95 ± 0.97	3.38 ± 1.10	3.08 ± 1.17

No. inadequate antimicrobials (mean ± SD)	1.88 ± 1.05	2 ± 0.58	1.65 ± 0.83	1.82 ± 0.91
Inadequate prescription upon discharge	24/32 (75)	4/8 (50)	11/25 (44)*	39/65 (60)
Duration of antimicrobial therapy, days, (median, IQR)	15 (11-17)	14 (10-16)	15 (12.5-17)	15 (11-17)
Duration of IV therapy, days, (median, IQR)	6 (3-9.5)	8 (4-14)	8 (4-15)	7 (4-12)
Duration of PO therapy, days, (median, IQR)	11 (8-13)	10 (7.3-12.8)	10 (6-14)	10 (7-13)
Consultation with an ID specialist	23 (45)	8 (42)	23 (58)	54 (49)
Time to effective antibiotics	2.7 (1.1-5.5)	1.6 (0-17.6)	1.7 (0.8-4.8)	2.2 (0.8-5.5)

Number (%). Abreviations : IQR : interquartile range, SD : standard deviation, IV : intravenous, PO : per os (orally), ID : infectious disease. * statistically significant differences (*P* value < 0.05), reference category = pre-APSS period.
